# Acute or Subacute, the Optimal Timing for Uncomplicated Type B Aortic Dissection: A Systematic Review and Meta-Analysis

**DOI:** 10.3389/fsurg.2022.852628

**Published:** 2022-05-03

**Authors:** Yang Yang, Xi-Hao Zhang, Zuo-Guan Chen, Yong-Peng Diao, Zhi-Yuan Wu, Yong-Jun Li

**Affiliations:** Department of Vascular Surgery, Beijing Hospital, National Center of Gerontology, Institute of Geriatric Medicine, Chinese Academy of Medical Sciences, Beijing, China

**Keywords:** TEVAR, uncomplicated type B aortic dissection, timing, endovascular treatment, endovascular aortic repair

## Abstract

**Objective:**

To evaluate the optimal timing (acute or subacute) of thoracic endovascular aortic repair (TEVAR) for uncomplicated B aortic dissection (uTBAD) through a systematic review and meta-analysis.

**Method:**

A comprehensive literature search was undertaken across three major databases (EMBASE/Medline, PubMed, and Cochrane Library) and was assessed until November 2021 to identify studies reporting the outcomes of TEVAR utilized to treat patients with uTBAD. The continuous variables were compared between the two groups using *t*-test and the categorical variables were compared using the χ^2^-test. A meta-analysis was used to produce pooled odds ratios for early and follow-up outcomes. The random effects models were applied. A statistical analysis was performed using R software v.4.1.

**Result:**

A comprehensive literature search found 490 citations published within the predetermined time span of the analysis. Three studies including 1,193 patients (acute group 718, subacute group 475) were finally included for downstream meta-analysis. An acute uTBAD group presented with higher rates both in 30-day complications (20.5 vs. 13.7%; *p* = 0.014) and mortality (4.6 vs. 1.3%; *p* = 0.004) than subacute group. The respiratory complications were significantly higher in the acute group than in the subacute group (10.8 vs. 5.0%; *p* = 0.015). The procedure success rate (90.8 vs. 93.6%; *p* = 0.329), the follow-up mortality (7.7 vs. 7.6%; *p* = 1) and dissection-related late mortality (3.9 vs. 5.3%; *p* = 0.603) showed no significant difference.

**Conclusion:**

Our meta-analysis suggested that despite significantly higher 30-day complications and 30-day mortality in the acute uTBAD group, there was no significant difference in the follow-up mortality between the two groups.

**Systematic Review Registration:**

PROSPERO, identifier: CRD42021247609.

## Introduction

Since 1999, endovascular stent–graft was introduced as a novel treatment option for patients with type B aortic dissection (TBAD) by Dake et al. (1) and Nienaber et al. (2), and it has now become the first choice for the treatment of acute complicated TBAD (cTBAD) according to recent guidelines (3–5). Uncomplicated TBAD (uTBAD) was historically managed medically with anti-impulse and anti-hypertensive therapy (6). Recently, more and more doctors began to advocate the treatment of uTBAD with thoracic endovascular aortic repair (TEVAR) (7–10). Lou and colleagues summarized that TEVAR treatment within 14 days provided the best chance for complete remodeling and it can reduce aortic-related mortality (11). The latest European society for vascular surgery (ESVS) guidelines suggest that the patients with uTBAD may benefit from TEVAR in subacute period (IIa, B) (5). It was mainly based on a reference that compared optimal medical treatment with TEVAR on patients suffering from uTBAD, instead of any references focusing on the optimal timing (acute vs. subacute) of TEVAR (12, 13) while other guidelines did not have specific recommendations on this issue. So, which is the optimal timing of TEVAR for patients with uTBAD? To this end, we performed a systematic review and meta-analysis to obtain the optimal timing of TEVAR for patients with uTBAD.

## Methods

### Study Protocol

The protocol was registered on the International Prospective Register of Systematic Reviews (PROSPERO) with the number of CRD42021247609. The analysis was performed according to the recommendations in the Preferred Reporting of Systematic Reviews and Meta-Analysis (PRISMA) statement (14). The analysis objectives were to investigate pre-operative characteristics, peri-operative (early) and post-operative (late) outcomes of patients undergoing TEVAR for uTBAD in acute vs. subacute period. The P.I.C.O. (patient: patients with uTBAD; intervention: TEVAR; comparison: acute vs. subacute period; outcome: 30-day complications and mortality et al.) model was used to select relevant articles (15).

### Data Sources

Three databases (EMBASE/Medline, PubMed, and Cochrane Library) were adopted in this study. The literature search strategy includes: (“stent” OR “endovascular”) AND (“DeBakey III” OR “type B”) AND “uncomplicated” AND “aortic dissection” AND (“timing” OR “phase” OR “period”) and were assessed until November 2021. Studies were identified if reporting the outcomes of TEVAR for patients with uTBAD. The searching evidence was limited to the English language and human studies.

### Study Selection Criteria and Data Extraction

**Figure 1:**
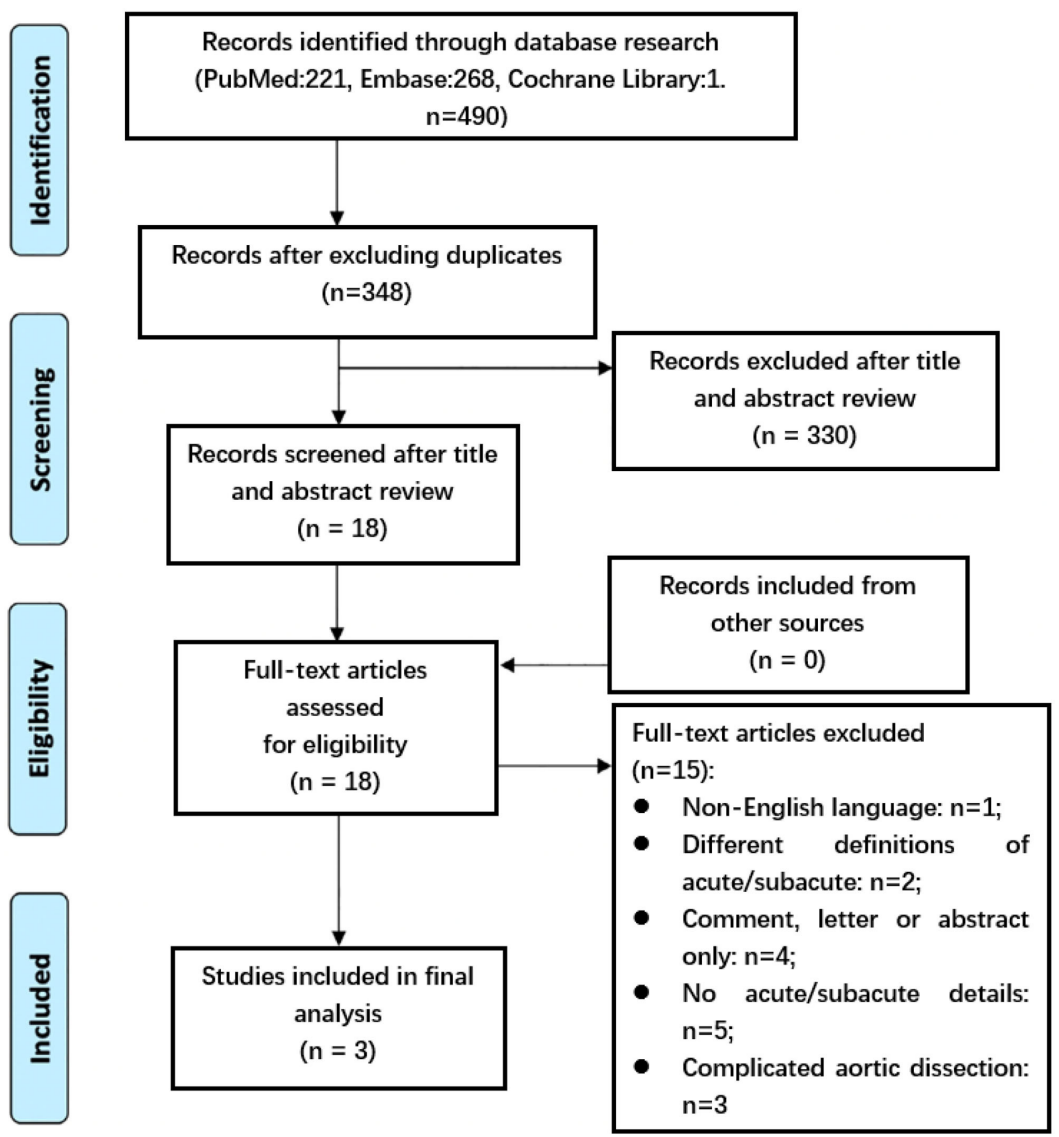
Preferred reporting items for systematic review and meta-analysis flow diagram detailing our search and selection process for the initial stages of the review. Acute = within the first 14 days from onset of symptoms. Subacute = beyond 14 days from onset of symptoms.

This review was conducted and reported according to the preferred reporting items of the systematic review and meta-analysis report published in 2009 (16). The selection criteria are as follows: Studies reporting outcomes in cohorts of more than 20 patients undergoing the TEVAR procedure and providing data for postoperative outcomes; studies that have compared the outcomes of TEVAR utilized to treat patients with acute uTBAD and subacute uTBAD. Exclusion criteria included removing papers based on study type, namely, case reports, cases series, single-arm studies, and literature reviews; studies that referred to type A dissections or to a combined hybrid endovascular or open thoracic aorta repair were excluded unless they included a subgroup of patients that were treated or further treated with TEVAR for a form of type B dissection; articles containing insufficient data <25% of predefined variables extractable) were excluded from the analysis; if various publications on the same population of patients were identified or if study populations overlapped then only the latest report was included unless the outcomes were mutually exclusive. After excluding duplicated citations, all titles and abstracts were reviewed by independent reviewers; the full-text of studies that met inclusion criteria were obtained and those were reviewed to extract data. Study data were extracted by another independent reviewers, and if necessary, a second was consulted to reach a consensus by rereviewing the full text of articles (Figure 1).

Each article was analyzed with respect to 41 predefined variables regarding clinical characteristics, procedural data, in-hospital, and long-term outcomes using a standardized protocol [see Appendix, as modified according to Eggebrecht's meta-analysis (17)]. Extraction of data was performed by the first authors and independently verified by co-authors. Unspecified information was classified as not available. As a result, the number of patients (denominator) varies with the specific variables reported in the analysis.

### Risk of Bias

**Figure 2:**
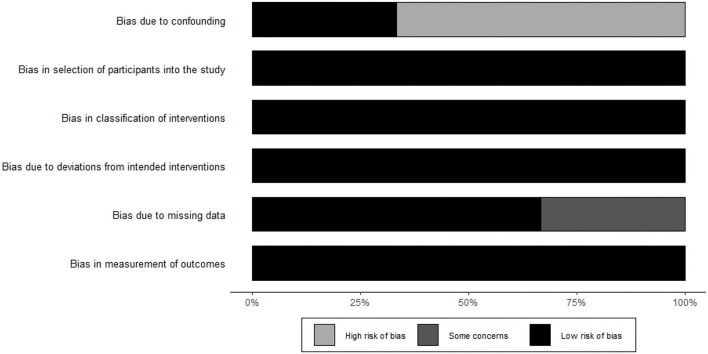
Risk of bias. Methodological quality assessment with the latest version of the ROBINS-I checklist for non-RCT. Gray = high risk of bias. Dark gray = some concerns. Black = low risk of bias.

The quality assessment was evaluated with the latest version of the ROBINS-I checklist for non-randomized studies (18). Non-randomized studies were judged for confounding bias, selection bias, bias in classification of interventions, bias in deviation from intended interventions, bias due to missing data, bias in measurement of outcome and bias in selection of the reported results. Each study was assigned a “low of risk,” “high of risk,” or “unclear” risk of bias (Figure 2).

### Definitions

TBAD was classified according to the Stanford classification. In this review, dissection was classified into two period including acute (within the first 14 days from onset of symptoms) and subacute (beyond 14 days from onset of symptoms). Thoracic stent–grafts placed in the TEVAR procedure were deployed retrograde *via* percutaneous femoral artery access employing the pre-closing technique. Procedural success was defined by the technically successful deployment of the endoprosthesis at the intended target location. Aortic-related death referred to death caused by aortic reasons, like aortic rupture. The complications occurred in hospital stay was classified into 30-day complications included aortic rupture, organ failure (renal failure and heart failure), heart complications (myocardial infarction and congestive heart failure), renal complications (renal ischemia and renal failure), respiratory complications, endoleak, neurological complications (spinal cord ischemia, paraplegia, and dialysis). A re-intervention was defined as the need for any surgical conversion or additional endovascular stent–graft procedures. The data that were not reported in the articles were recorded as “n.a.”

As with the included studies, two groups were analyzed in this study. The patients with acute uTBAD were referred to acute group and those with subacute uTBAD were included in subacute group.

### Statistical Analysis

The continuous variables were compared between the two groups using *t*-test and the categorical variables were compared using the χ^2^-test. The random effects model was used to evaluate the results. The proportion was compared between the two groups to see if there was an overlap of 95% confidence intervals (CI) to assess statistical significance. Statistical analysis was performed using R software v.4.1.

## Result

### Study Selection

Comprehensive literature search resulted in 490 citations published within the predetermined time span of the analysis. Thereafter, 18 studies' full-text were assessed for eligibility after excluding duplicates and studies that have little correlation with our purpose. Of these, one was excluded for non-English language; four were excluded for comment, letter or abstract only; five were excluded for no acute or subacute details; three were excluded for about cTBAD. In addition, two of the studies were excluded for different definitions of acute and subacute period. Wang et al. (22) considered dissection as an acute event if it occurred within the first 30 days from onset of symptoms. And Schwartz et al. (23) stratified timing of intervention into early (within 180 days of initial presentation) and late (181 days and later). All three articles were non-randomized, retrospective studies. The total number of patients included in the analysis was 1,193 and data was extracted from three studies (19–21). Among them, 718 patients were categorized as acute uTBAD and 475 as subacute uTBAD. The major information of each study including patients, procedure success, emergency conversion, 30-day complications and so on are presented in Table 1.

**Table 1:** Detailed overview over the analyzed reports.

**References**	**Year**	**Phase**	**Patients (n)**	**Procedure success (n)**	**Emergency conversion (n)**	**30-day complications (n)**	**Organ failure (n)**	**Postoperative endoleak** **(n)**	**30-day neurological complications (n)**	**Paraplegia** **(n)**	**30-day mortality (n)**	**Late reintervention (n)**	**Late aortic rupture (n)**	**Late mortality (n)**
Xiang et al. (19)	2021	Acute	142	128	n.a	25	5	14	1	0	2	n.a	n.a	10
		Subacute	96	91	n.a	9	4	5	0	0	0	n.a	n.a	9
Xie et al. (20)	2021	Acute	130	119	0	19	n.a	11	4	0	5	6	2	5
		Subacute	137	127	0	15	n.a	10	4	0	1	2	5	11
Torrent et al. (21)	2021	Acute	446	n.a	0	103	7	n.a	n.a	n.a	26	45	n.a	39
		Subacute	242	n.a	0	41	2	n.a	n.a	n.a	5	19	n.a	14

*Details of included studies of systematic literature review and meta-analysis of outcomes of patients with acute and subacute uncomplicated type B aortic dissection (uTBAD) treated by thoracic endovascular aortic repair (TEVAR). Complications occurred in hospital stay was classified into 30-day complications included aortic rupture, organ failure (renal failure and heart failure), heart complications (myocardial infarction and congestive heart failure), renal complications (renal ischemia and renal failure), respiratory complications, endoleak, neurological complications (spinal cord ischemia, paraplegia and dialysis)Data not reported in the articles are recorded as n.a*.

### Patient Characteristics

The characteristics of the selected patient population are shown in Table 2. Patients undergoing interventions for acute uTBAD and subacute uTBAD were of similar age (*p* = 0.792) and sex (*p* = 0.186). There was no difference in the diabetes mellitus, hypertension, chronic obstructive pulmonary disease, coronary artery disease, cerebrovascular disease, renal insufficiency between both groups(*p*>0.05).

**Table 2:** Patient characteristics.

	**Acute (*n*/*n*)**	**Subacute (*n*/*n*)**	***p*** **-value**
Male gender	504/718 (70.2%)	351/475 (73.9%)	0.186
Baseline characteristics			
Smoking	283/718 (39.4%)	162/475 (34.1%)	0.073
Hypertension	599/718 (83.4%)	390/475 (82.1%)	0.607
Coronary artery disease	83/718 (11.6%)	60/475 (12.6%)	0.641
Cerebrovascular disease	41/718 (5.7%)	32/475 (6.7%)	0.548
Renal insufficiency	21/272 (7.7%)	17/233 (7.3%)	0.969
Chronic pulmonary disease	95/718 (13.2%)	49/475 (10.3%)	0.155
Diabetes mellitus	66/718 (9.2%)	48/475 (10.1%)	0.671

*Patient characteristics of included studies of systematic literature review and meta-analysis of outcomes of patients with acute and subacute uncomplicated type B aortic dissection (uTBAD) treated by thoracic endovascular aortic repair (TEVAR)*.

### Procedural Data and In-Hospital Course

**Figure 3:**
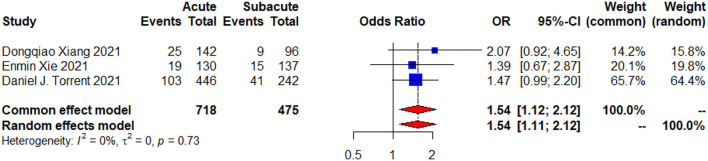
A 30-day complications forest plot of comparison of TEVAR for patients with acute vs. subacute uTBAD. The blue squares denote the OR or risk differences, the horizontal lines represent the 95% CI) and the red diamond denotes the pooled effect size. OR, odds ratio; CI, confidence interval.

**Figure 4:**
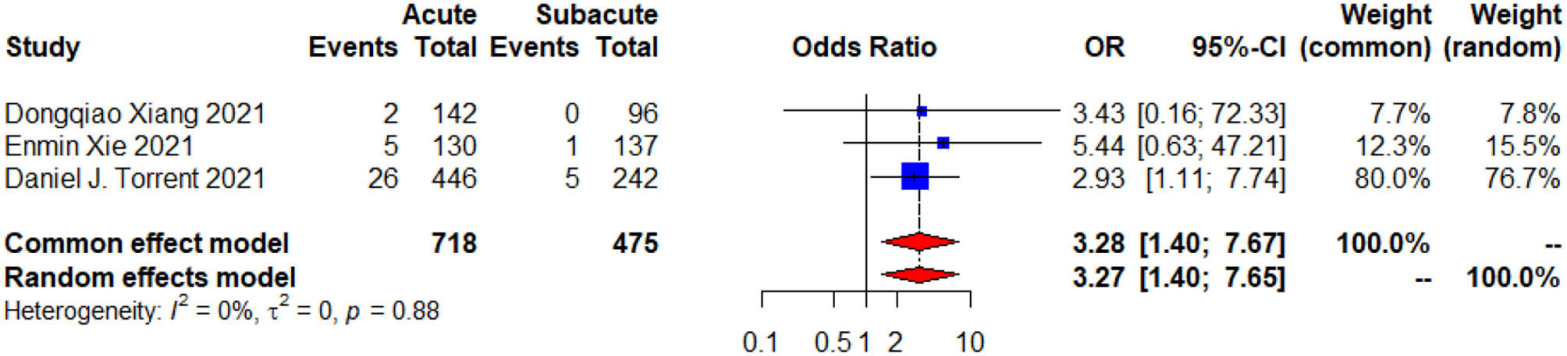
A 30-day mortality Forest plot of comparison of TEVAR for acute vs. subacute uTBAD. The blue squares denote the odds ratios or risk differences, the horizontal lines represent the 95% confidence intervals (CI), and the red diamond denotes the pooled effect size. OR, odds ratio; CI, confidence interval.

The procedural success was obtained in 90.8% of patients with acute uTBAD and 93.6% of patients with subacute uTBAD (*p* = 0.329, Table 3). In addition, both groups of the patients did not receive emergency surgical conversion during hospital. There was a significantly higher proportion of in-hospital complications in the patients with acute uTBAD as compared to the patients with subacute uTBAD (20.5 vs. 13.7%; *p* <0.05; Figure 3). Within all of the in-hospital complications that reported by these studies, the incidence of respiratory complications was higher in the patients with acute uTBAD (10.8 vs. 5.0%; *p* <0.05), while no difference was observed in other in-hospital complications (Table 3). Within the 30-day period, the patients with acute uTBAD presented a significantly higher proportion of mortality (4.6 vs. 1.3%; *p* <0.05; Figure 4).

**Table 3:** Procedural data and in-hospital course.

	**Acute (*n*/*n*)**	**Subacute (*n*/*n*)**	***p*** **-value**
More than one stent–graft placed	11/130 (8.5%)	18/137 (13.1%)	0.303
Procedure success	247/272 (90.8%)	218/233 (93.6%)	0.329
Emergency conversion	0/576 (0%)	0/379 (0%)	n.a
30-day complications	147/718 (20.5%)	65/475 (13.7%)	0.014
Aortic rupture	4/272 (1.5%)	0/233 (0%)	0.175
Organ failure	12/588 (2.0%)	6/338 (1.8%)	0.972
Heart complications	47/446 (10.5%)	20/242 (8.3%)	0.409
Renal complications	3/142 (2.1%)	1/96 (1.0%)	0.907
Respiratory complications	48/446 (10.8%)	12/242 (5.0%)	0.015
Type I endoleak	25/272 (9.2%)	15/233 (6.4%)	0.329
30-day neurological complications	12/718 (1.7%)	13/475 (2.7%)	0.293
Spinal cord ischemia	12/576 (2.1%)	13/379 (3.4%)	0.285
Paraplegia	0/142 (0%)	0/96 (0%)	n.a.
Cerebrovascular disease	20/718 (2.8%)	6/475 (1.3%)	0.119
30-day mortality	33/718 (4.6%)	6/475 (1.3%)	0.004
Aorta-related mortality	1/142 (0.7%)	0/96 (0%)	1
Non-aorta-related mortality	1/142 (0.7%)	0/96 (0%)	1

*Procedural data and in-hospital course of included studies of systematic literature review and meta-analysis of outcomes of patients with acute and subacute uncomplicated type B aortic dissection (uTBAD) treated by thoracic endovascular aortic repair (TEVAR)*.

### Follow-Up Data

**Figure 5:**
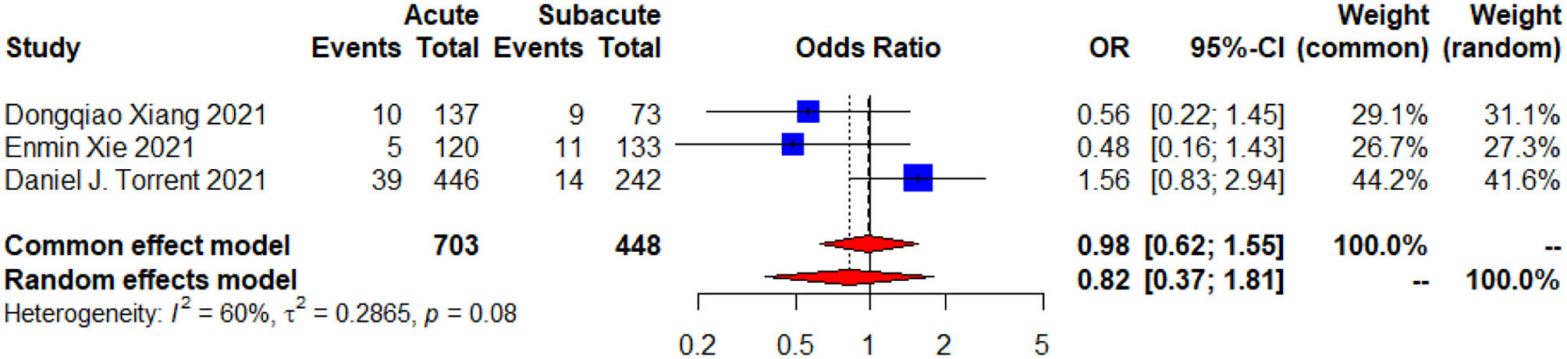
The follow-up mortality forest plot of comparison of TEVAR for patients with acute vs. subacute uTBAD. The blue squares denote the OR or risk differences, the horizontal lines represent the 95% CI, and the red diamond denotes the pooled effect size. OR, odds ratio; CI, confidence interval.

Regarding the follow-up data, two of these articles (19, 21) offered 1 year follow-up data,and two (19, 20) provided their follow-up data of more than 3 years. No significant difference can be concluded in late mortality (Figure 5), late reintervention, late complications and aortic rupture during follow-up between the two groups (*p* > 0.05; Table 4).

**Table 4:** Follow-up data of TEVAR for patients with acute vs. subacute uTBAD.

	**Acute (*n*/*n*)**	**Subacute (*n*/*n*)**	***p*** **-value**
Follow-up mortality	54/703 (7.7%)	34/448 (7.6%)	1
Dissection-related late mortality	10/257 (3.9%)	11/206 (5.3%)	0.603
Non-dissection-related late mortality	4/257 (1.6%)	8/206 (3.9%)	0.203
Late reintervention	51/566 (9.0%)	21/375 (5.6%)	0.072
Late complications	43/250 (17.2%)	30/214 (14.0%)	0.418
Aortic rupture during follow-up	2/120 (1.7%)	5/133 (3.8%)	0.529

*Follow up data of included studies of systematic literature review and meta-analysis of outcomes of patients with acute and subacute uncomplicated type B aortic dissection (uTBAD) treated by thoracic endovascular aortic repair (TEVAR). Follow up data was collected over 30 days after surgery*.

## Discussion

Ever since the Food and Drug Administration (FDA) broadly approved TEVAR for the treatment of aortic pathologic processes in 2013; TEVAR for uncomplicated dissection had become relatively common despite BMT only (24–26). Several studies have shown that TEVAR for TBAD resulted in similar aortic remodeling, clinical outcomes, and procedure-related complications in both acute and subacute periods (27, 28). The latest guidelines provided some suggestions, but did not clarify which period was better with TEVAR only. Some clinical centers have presented their experience on the timing of TEVAR for uTBAD (19–23). To this end, our team aimed to perform a system review and meta-analysis on the optimal timing of TEVAR for uTBAD. However, our study stated that TEVAR performed in acute uTBAD groups did not bring as much profile as subacute groups in early outcomes, without significant difference in late outcomes.

The 30-day complications (Figure 3) and mortality (Figure 4) were evaluated by the random effects model and presented with low heterogeneity (*p* = 0.73, *I*^2^ = 0%; *p* = 0.88, *I*^2^ = 0%). The results of χ^2^-test (Table 3) showed increased risk of early outcomes in acute period (*p* < 0.05). When we further analyzed these data, we noticed that the incidence of most complications showed a higher tendency in acute group, especially the respiratory complications (*p* = 0.015; Table 3). Although no significant differences were found in the baseline characteristics of both groups, we found that the smoking rate was higher in the acute group (39.4 vs. 34.1%, *p* = 0.073). Whether the cigarette-influenced patients' respiratory system and caused the respiratory complications or not, further studies needed to be designed. Besides, another trend was also observed at the seemingly higher rates of chronic pulmonary disease in the acute group (*p* = 0.155). Both factors may carry higher risks for the respiratory complications after TEVAR. However, further studies were required to prove this observation.

The current review noticed that TEVAR in acute uTBAD groups did not bring as much profile as subacute groups in early outcomes. This was consistent with a previous report analyzing the relationship between the timing of TEVAR and outcomes in TBAD, which showed that 30-day mortality were higher in acute period (17.5%) than that in the subacute period (0%) (29). However, acute intervention potentially owns an advantageous choice, because the dissection flap is most pliable and provides the best chance for complete remodeling (11). And favorable remodeling can also reduce the likelihood of aneurysmal degeneration and aorta-related mortality (12). However, this advantage must be balanced with the increased risk of 30-day complications and mortality.

Regarding the follow-up mortality (Figure 5) between the two groups, the common effect model and random effect model (*p* = 0.08, *I*^2^ = 60%) resulted in heterogeneities. This may due to the different follow-up periods as Torrent et al. (21) reported follow-up data at 1 year, while Xie et al. (20) and Xiang et al. (19) reported follow-up data at more than 3 years. However, despite the high heterogeneities, data from all three studies proposed that there were no statistically significant differences on long-term outcomes. To better evaluate the prognosis of patients, we need more follow-up data, and furthermore, follow-up data of the same patient group at different time nodes.

Our analysis showed that late reintervention in the acute group (9.0%) was higher than in the subacute group (5.6%), with no significant differences (*p* = 0.072). However, the potential risk is not only related to the timing of TEVAR, but also related to many other relevant risk factors. A recent review summarized some high-risk radiological features of uTBAD: an initial false lumen (FL) diameter of ≥ 22 mm, a maximum aortic diameter of ≥ 40 mm at initial presentation, a patent or partially thrombosed false lumen, and an initial entry tear of ≥ 10 mm (30). In addition, Dong et al. (31) reported that these risk factors could predict the reintervention after TEVAR in patients with TBAD. Thus, we need to consider whether the patients with acute uTBAD without risk factors should accept TEVAR as soon as possible.

## Limitation

There are several limitations that should be acknowledged here in this manuscript. First, the studies included were all retrospective analysis that reflects a single-center experience. Second, the absence of available randomized controlled studies left us with a low level of evidence. So far, due to the small sample size, it was hard to analyze the heterogeneity and brought bias. Further, only short- and mid-term follow-up data are presented in all studies while long-term outcomes need to be evaluated.

## Conclusion

This meta-analysis suggests that 30-day complications and 30-day mortality were higher in the patients with acute uTBAD group, but no significant difference was observed in the follow-up mortality between the two groups.

## Data Availability Statement

The original contributions presented in the study are included in the article/Supplementary Material, further inquiries can be directed to the corresponding author.

## Author Contributions

Y-JL and Z-YW contributed to conception and design of the study. YY and X-HZ organized the database. YY and Z-GC performed the statistical analysis. X-HZ, Y-PD, and YY wrote the first draft of the manuscript. X-HZ, Z-YW, Z-GC, Y-PD, and Y-JL revised the manuscript. All authors contributed to manuscript revision, read, and approved the submitted version.

## Funding

This study was supported by Beijing Hospital Clinical Research 121 Project (BJ-2018-089) and National Key Research and Development Project of China (2018YFC2000301 and 2020YFC2008003).

## Conflict of Interest

The authors declare that the research was conducted in the absence of any commercial or financial relationships that could be construed as a potential conflict of interest.

## Publisher's Note

All claims expressed in this article are solely those of the authors and do not necessarily represent those of their affiliated organizations, or those of the publisher, the editors and the reviewers. Any product that may be evaluated in this article, or claim that may be made by its manufacturer, is not guaranteed or endorsed by the publisher.
