# Supplementary material for: Acute or Subacute, the Optimal Timing for Uncomplicated Type B Aortic Dissection: A Systematic Review and Meta-Analysis
Source: Front Surg. 2022 May 3;9:852628. doi: 10.3389/fsurg.2022.852628 (PMC9110682; doi:10.3389/fsurg.2022.852628)
Supplement: Supplementary file 1 [file Table_1.DOCX]

# Appendix

## List of variables abstracted from each article (if available)

### Patient characteristic

Number of patients with dissection

Age

Gender

Hypertension

Coronary artery disease

Cerebrovascular disease

Renal insufficiency

Chronic obstructive pulmonary disease

Diabetes mellitus

### Procedural data

Number of stent-grafts per patient

Stent-graft diameter

Stent-graft length

Procedure success

Emergency conversion

### In-hospital data

30-day complications

Aortic rupture

Organ failure

Type I endoleak

Myocardial complications

Renal complications

Pulmonary complications

Overall neurologic complications

Spinal cord ischemia

Paraplegia

Stroke

Need for early surgical conversion

Adjunctive endovascular procedure

Length of hospital stay

30-day mortality

Aorta-related mortality

Non-aorta related mortality

### Follow-up data

Duration of follow-up

Late reintervention

Late complications

Late neurologic complications

Late retrograde type A-dissection

Aortic rupture during follow-up

False lumen thrombosis

Follow-up mortality

Aorta-related late mortality

Non-aorta related late mortality
